# *Anaplasma phagocytophilum* Variant 1 from White-Tailed Deer (*Odocoileus virginianus*) in Feeding *Ixodes scapularis* Larvae

**DOI:** 10.3390/pathogens15090933

**Published:** 2026-09-04

**Authors:** Patrick Pearson, Mileena Ryan, Martha B. Leo, Guang Xu, Martin J. R. Feehan, Dowd Naik, Melissa A. Prusinski, Stephen M. Rich

**Affiliations:** 1 Laboratory of Medical Zoology, Department of Microbiology, University of Massachusetts, Amherst, MA 01003, USA; pbpearson@umass.edu (P.P.); mileenaryan@umass.edu (M.R.); mbleo@umass.edu (M.B.L.); gxu@umass.edu (G.X.); 2 New England Center of Excellence in Vector-Borne Diseases (NEWVEC), University of Massachusetts, Amherst, MA 01003, USA; 3 Massachusetts Division of Fisheries and Wildlife, Westborough, MA 01581, USA; martin.feehan@mass.gov; 4 Bureau of Communicable Disease Control, New York State Department of Health, Albany, NY 12237, USA; dowd.naik@health.ny.gov (D.N.); melissa.prusinski@health.ny.gov (M.A.P.)

**Keywords:** *Anaplasma phagocytophilum*, *Ixodes scapularis*, white-tailed deer, variants

## Abstract

*Anaplasma phagocytophilum* is a tick-borne bacterium and the causative agent of human granulocytic anaplasmosis. Two major variants have been identified in North America: a human pathogenic variant (Ap-ha) that is maintained by small mammals and a nonpathogenic variant (Ap-v1) that is maintained by white-tailed deer (*Odocoileus virginianus*). Despite white-tailed deer being recognized as the main reservoir of Ap-v1, few studies have directly demonstrated transmission of Ap-v1 from white-tailed deer to feeding *I. scapularis* ticks, and transmission to feeding larvae remains undescribed. In this study, we collected samples from an outdoor white-tailed deer research facility during an *I. scapularis* larval questing season in the eastern United States to investigate the variants infecting white-tailed deer and acquisition by feeding larvae. Although both variants were present in questing ticks onsite, white-tailed deer were persistently infected with only Ap-v1 and transferred this variant to feeding *I. scapularis* larvae with high efficiency. These results support the concept that white-tailed deer play a role in the maintenance of Ap-v1 in nature and highlight the utility of captive white-tailed deer research facilities for studying vector–pathogen–host biology.

## 1. Introduction

*Anaplasma phagocytophilum* is the causative agent of human granulocytic anaplasmosis and is transmitted by ticks of the genus *Ixodes*, such as *I. scapularis* in the eastern United States [1,2]. Based on variation at the 16S rDNA locus, two major *A. phagocytophilum* variants have been described in North America: a human-active variant (Ap-ha) and another referred to as variant 1 (Ap-v1) [3,4,5,6]. The Ap-ha variant is pathogenic to humans and is thought to be maintained primarily by small mammal reservoirs, particularly the white-footed mouse (*Peromyscus leucopus*) [4,7]. In contrast, the Ap-v1 variant is not known to cause human disease and has white-tailed deer (*Odocoileus virginianus*) as its primary reservoir [4,8,9].

Although white-tailed deer (WTD) are recognized as the principal reservoir of Ap-v1, direct evidence of transmission of Ap-v1 from WTD to feeding ticks remains limited. Previous work has demonstrated that *I. scapularis* nymphs feeding on artificially infected WTD can acquire Ap-v1 and retain infection after molting to the adult stage [8]. A field study reported an unusually high percentage of Ap-v1 in female *I. scapularis* collected from naturally infected WTD, suggesting acquisition of Ap-v1 during feeding [9]. However, to the best of our knowledge, evidence of Ap-v1 transmission to feeding *I. scapularis* larvae is lacking. This gap may reflect the logistical difficulty of collecting engorged larvae from WTD, as the *I. scapularis* larval questing period precedes the typical WTD hunting season in the northeastern United States. In this report, we analyzed both on- and off-host ticks from a captive WTD study site collected during the *I. scapularis* larval questing period to determine *A. phagocytophilum* infection in the WTD, quantify acquisition by feeding larvae from infected WTD, and identify circulating *A. phagocytophilum* variants.

## 2. Materials and Methods

The captive WTD used in this study were maintained in the Cervid-Vector Ecology Research Station (C-VERS). C-VERS is a ~2-hectare, outdoor, fenced research facility located on forested property owned by the University of Massachusetts (Amherst, MA, USA). C-VERS is permitted to maintain a group of 15 WTD, though the population of captive WTD is dynamic, as new rehabilitated fawns that cannot be released back into the wild are added annually as part of a collaboration with the Massachusetts Division of Fisheries and Wildlife (MassWildlife). The WTD used in this study were placed at C-VERS as fawns in July 2024 and maintained under the same husbandry and environmental conditions. The WTD maintained at C-VERS are naturally parasitized year-round by a local, established population of *I. scapularis* ticks and exposed to pathogens they may be harboring. All procedures involving the WTD were performed in accordance with an approved University of Massachusetts Amherst Institutional Animal Care and Use Committee (IACUC) protocol (IACUC protocol #5163) and under a permit issued by MassWildlife (permit #046.25LP).

Larval *I. scapularis* ticks and blood samples were collected from three female, >1-year-old WTD on 23 July, 20 August, and 17 September 2025. These were the only WTD present at C-VERS for the first sampling date and additional animals received later in the year were excluded from the study. Since this was an observational, small-scale study using a limited number of captive WTD, no control group or randomization was used, and the results should be considered preliminary. The WTD were chemically immobilized using a combination of ~166 mg Telazol (tiletamine and zolazepam) and ~200 mg xylazine injected either by pole syringe or via a remote drug delivery device, and then whole blood was collected into EDTA-treated tubes from the cephalic vein. Actively feeding larval ticks were opportunistically collected from each WTD while the animals were immobilized during blood draws. Larvae were manually removed using tweezers and immediately placed into plastic vials. Adult WTD-collected *I. scapularis* ticks are not reported here, since they were not always collected or analyzed and the focus of this study was larval acquisition of *A. phagocytophilum*. Questing larval and adult *I. scapularis* ticks were collected by flagging vegetation around C-VERS from August through November 2025. All field- and WTD-collected ticks in this study were initially identified as *Ixodes* spp. using a dissecting microscope as previously described [10], individually transferred to 2.0 mL tubes, and stored at −80 °C prior to nucleic acid extraction. Blood samples were also stored in 0.5 mL aliquots at −80 °C prior to nucleic acid extraction. A subset (*N* = 12) of the WTD-fed, larval ticks was initially placed in an environmental chamber at 23 °C, ~85% humidity, and a 12-h light/12-h dark cycle to encourage molting before the ticks were prepared for nucleic acid extraction.

Total nucleic acid was extracted from the tick and blood samples using the MasterPure Complete DNA and RNA Purification Kit (LGC Biosearch Technologies, Guildford, UK) following the manufacturer’s protocols. The total nucleic acid extracts from the ticks were tested by two real-time PCR assays to confirm *Ixodes* tick species and detect the presence of *A. phagocytophilum* DNA as previously described [10]. The first real-time PCR assay targets the *ITS* gene of *I. scapularis* and the second assay targets the *msp2* gene of *A. phagocytophilum* [10]. Every tick used in this study was confirmed to be *I. scapularis* by this method. The presence of *A. phagocytophilum* DNA in the blood samples was determined using the same real-time PCR assay targeting the *msp2* gene. Samples positive for *A. phagocytophilum* were further characterized by a SNP genotyping assay that can simultaneously detect the Ap-ha and Ap-v1 variants in a sample [5], with modifications as previously described [6]. The individuals performing the initial in-processing, nucleic acid extraction, and real-time PCR were not blinded to the sample origin, type, or infection status. However, the individuals performing the SNP genotyping were effectively blinded regarding sample description and history. No formal statistical tests were performed, and the results are presented as raw counts, percentages, and 95% confidence intervals calculated in GraphPad Prism (version 11.0.2).

## 3. Results

*A. phagocytophilum* DNA was detected in every WTD at each time point and the variant was determined to be Ap-v1 (Table 1). No mixed genotype infections were found in any of the WTD blood samples. In total, 43 fed *I. scapularis* larvae were collected from the WTD over the course of the three time points, with 21 larvae collected from one deer, 17 from another, and 5 from the third deer. Only 1 of the 12 larvae placed in the environmental chamber molted to a nymph on day 38, and the remaining larvae (*N* = 11) were subsequently removed on day 72 and tested as described above. The molted nymph was negative for *A. phagocytophilum*. Of the remaining WTD-fed larvae, all (42/42) were infected with *A. phagocytophilum* and Ap-v1 was the only variant detected in 39 of the 42 positive samples (Table 1). The *A. phagocytophilum* variant could not be determined in the other 3 samples.

Questing *I. scapularis* larvae (*N* = 103) and adults (21 females and 30 males; *N* = 51 total) were collected from vegetation around C-VERS from August through November of 2025. The *A. phagocytophilum* infection prevalence in field-collected *I. scapularis* larvae and adults was 0% (0/103) and 14% (7/51), respectively (Table 1). In the adult *I. scapularis* samples positive for *A. phagocytophilum*, two males were infected with Ap-v1 and five adults (four males and one female) were infected with Ap-ha. No mixed genotype infections were detected in any of the adult *I. scapularis* samples.

## 4. Discussion

These results support the role of WTD as competent reservoirs for the Ap-v1 variant of *A. phagocytophilum*. In this study, the three WTD were persistently infected with *A. phagocytophilum* throughout the study period, and the infecting variant was exclusively Ap-v1. Since the WTD had been maintained at an outdoor facility in Massachusetts since July 2024, they were subjected to multiple *I. scapularis* nymph and adult questing seasons and were likely repeatedly exposed to *A. phagocytophilum* prior to our study period in 2025. The repeated exposures to *A. phagocytophilum*, and the fact that WTD can remain infected for up to 63 days following artificial inoculation with Ap-v1 [8], likely contributed to the persistent infections observed in the research animals. These results also suggest that wild WTD may be persistently infected in areas where there is active transmission of Ap-v1 among ticks and WTD. Interestingly, the Ap-ha variant was not detected in any of the WTD, even though it was present in questing adult *I. scapularis* ticks collected from the same environment. These results suggest that exposure to Ap-ha likely occurred and support previous research indicating that WTD are resistant to stable infection by the Ap-ha variant [9].

This study provides the first observation of Ap-v1 acquisition by feeding *I. scapularis* larvae from infected WTD. Every fed *I. scapularis* larva was infected with *A. phagocytophilum* and the genotyping assay determined that nearly all (39/42) were infected only with Ap-v1, suggesting efficient transfer of this variant from infected WTD to feeding larvae. The lack of infection with *A. phagocytophilum* in field-collected questing *I. scapularis* larvae is in agreement with previous studies showing no evidence of transovarial transmission of *A. phagocytophilum* [11,12] and indicates that the infections in fed larvae were derived from WTD. The three samples for which the variant could not be determined may represent 16S rDNA variants that cannot be discerned using the SNP genotyping assay [4]. However, it is more likely that these results were simply due to low template concentration in the extracted sample and/or differences in sensitivity between the initial real-time PCR assay that detected *A. phagocytophilum* and the subsequent SNP genotyping assay. As a comparison, a recent study demonstrated that infected eastern chipmunks (*Tamias striatus*) and mice (*Peromyscus* spp.), in which only the Ap-ha variant was detected, transmitted infection to a mean of 52.9% (CI95: 16.7–89.1) and 36.8% (CI95: 24.9–48.7) of feeding larvae, respectively [13]. Our results indicate that transfer of Ap-v1 from infected WTD to feeding *I. scapularis* larvae is potentially more efficient. However, since only *A. phagocytophilum* DNA was detected by real-time PCR, it cannot be concluded that the bacteria were viable in the WTD-fed larvae or that successful colonization would occur. Furthermore, the transstadial transmission rate of Ap-v1 from WTD-fed *I. scapularis* larvae to nymphs could not be determined. Only 1 of 12 larvae successfully molted to a nymph and that tick was negative for *A. phagocytophilum*. Future work will be aimed at testing larger numbers of *I. scapularis* nymphs fed on infected WTD as larvae to determine the proportion that retain Ap-v1 infection after molting. 

A limitation of this study is that we did not perform a comprehensive genetic analysis of the collected *A. phagocytophilum*-positive samples, since the SNP genotyping assay only distinguishes two major North American variants by targeting the 16S rDNA locus. Other genetic targets such as *groEL, ankA,* and *sdhC* have been analyzed to gain a finer phylogenetic resolution and explore host associations of *A. phagocytophilum* [14,15,16]. Interestingly, several ecotypes of *A. phagocytophilum* have been described in Europe based on the *groEL* gene, with one ecotype strongly associated with roe deer (*Capreolus capreolus*) [14]. A recent study demonstrated that the Ap-ha variant clusters within the European host-generalist ecotype [17], but the relationship between our Ap-v1-positive samples and the European cervid-associated ecotypes remains unclear. Further characterization of our *A. phagocytophilum*-positive samples based on additional gene targets would help resolve where they fit into the larger *A. phagocytophilum* genetic framework. 

The current research clearly demonstrates the utility of captive WTD facilities for studying the basic biology and ecology of vectors and the pathogens they transmit. Previous research identifying *A. phagocytophilum* in naturally exposed WTD and parasitizing ticks typically relied on collections obtained during the WTD hunting season or controlled hunts [9,18,19,20]. While these collections are valuable, they are limited to certain periods of the year, do not allow for repeat sampling from individual WTD, and in the northeastern United States, they predominantly occur during the fall hunting season when only adult *I. scapularis* are active. Access to captive WTD maintained in a semi-natural habitat allows repeat sampling from multiple WTD at any point during the year to research host–pathogen–vector dynamics. These results also add to a growing number of studies demonstrating subadult *I. scapularis* ticks feeding on WTD [21,22,23]. Although WTD are the preferred host for adult *I. scapularis*, the role WTD play in feeding juvenile *I. scapularis* is potentially underestimated and warrants further study.

## Figures and Tables

**Table 1:** *A. phagocytophilum* infection prevalence and variants detected in WTD blood and *I. scapularis* ticks.

Sample Type	*A. phagocytophilum* Prevalence		Variants Detected		
	No. Positive/Total	% (CI 95)	Variant	No. Positive/No. Genotyped	% (CI 95)
WTD blood	9/9	100 (70.1–100.0)	Ap-v1	9/9	100 (70.1–100.0)
WTD-fed *I. scapularis* larvae	42/42	100 (91.6–100.0)	Ap-v1	39/42	93 (81.0–97.5)
			Undetermined	3/42	7 (2.5–19.0)
Field-collected *I. scapularis* larvae	0/103	0 (0.0–3.6)	N/A	N/A	N/A
Field-collected *I. scapularis* adults	7/51	14 (6.8–25.7)	Ap-v1	2/7	29 (5.1–64.1)
			Ap-ha	5/7	71 (35.9–94.9)

Note: Three blood samples collected per deer over three time points for a total of 9 samples.

## Data Availability

The data supporting the conclusions of this study will be made available by the authors upon request.

## Abbreviations

The following abbreviations are used in this manuscript:

WTD	White-tailed deer
Ap-ha	*Anaplasma phagocytophilum* human-active
Ap-v1	*Anaplasma phagocytophilum* variant 1
C-VERS	Cervid-Vector Ecology Research Station
PCR	Polymerase chain reaction
